# Long-Term Speech Outcomes in Unilateral Cleft Lip and Palate: A Comparative Study of Early and Delayed Hard Palate Closure

**DOI:** 10.1097/SCS.0000000000011975

**Published:** 2025-09-23

**Authors:** V.L. van Roey, L. Hofman, P.A.J. van der Goes, H.G. Poldermans, S. J. Haverkamp, E.C. Paes, A.B. Mink van der Molen, I.M.J. Mathijssen, S.L. Versnel

**Affiliations:** * Department of Plastic and Reconstructive Surgery, Sophia Children’s Hospital, Erasmus University Medical Centre, Rotterdam; † Department of Plastic and Reconstructive Surgery, Wilhelmina Children’s Hospital, University Medical Centre Utrecht, Utrecht; ‡ Department of Otorhinolaryngology and Head and Neck Surgery, Sophia Children’s Hospital, Erasmus Medical Centre, Rotterdam; § Speech and Language Therapy, Wilhelmina Children’s Hospital, University Medical Centre Utrecht, Utrecht, The Netherlands

**Keywords:** Hard cleft palate, ICHOM, longterm speech outcomes, palatoplasty, prospective study, speech assessment, speech disorders, Unilateral cleft lip and palate, articulation, velopharyngeal function

## Abstract

**Background:**

Evidence on the comparative effectiveness of surgical protocols for cleft lip and palate remains limited, especially regarding long-term speech outcomes. Therefore, this study evaluates speech outcomes at 5, 12, and 22 years in patients with unilateral cleft lip and palate (UCLP) to guide protocol selection.

**Methods:**

This prospective cross-sectional study included 285 UCLP patients treated at 2 Dutch academic hospitals. Patients were assessed at 1 of 3 predefined ages (5, 12, or 22 y) during routine follow-up; only a minority were assessed at more than one time point. Four protocols with different timing for hard palate closure were compared: Late Delayed Hard Palate Closure Protocol (L-DHPCP), Early Delayed Hard Palate Closure Protocol (E-DHPCP), One-Stage Palatoplasty Protocol (OSPP), and Oslo Protocol (OP). Speech outcomes were assessed using the ICHOM standard set for cleft lip and palate, including clinical (Percent Consonant Correct, Velopharyngeal Competence) and patient-reported (Intelligibility in Context Scale, CLEFT questionnaire) outcome measures and compared using ordinal logistic regression.

**Results:**

At 5 years, OSPP showed the most favourable speech results, significantly outperforming L-DHPCP and E-DHPCP. At 12 years, OP exhibited the most favourable results, while differences between protocols diminished. By 22 years, no significant differences were observed between the available protocols (E-DHPCP and L-DHPCP), though speech errors persisted in some patients.

**Conclusion:**

Early hard palate closure protocols, particularly OSPP and OP, were associated with better short-term and intermediate speech outcomes compared with DHPCPs. While differences diminished by 22 years, early closure should be prioritised in nonsyndromic UCLP patients to prevent persistent speech errors and minimise the burden of speech-enhancing surgery and speech therapy.

Over the years, various surgical protocols have been developed to address the challenges posed by a unilateral cleft lip and palate (UCLP). These protocols aim to optimize speech, maxillofacial growth, and nasolabial appearance while minimizing functional impairments and complications. A key area of variation among these protocols lies in the timing of hard palate and alveolar closure.^1^ Early hard palate closure has been associated with better speech outcomes. For instance, the Scandcleft trials demonstrated that early closure (12 mo) resulted in better articulation and resonance compared with delayed closure (36 mo) without an increase in complication rates.^2^ To further explore differences between protocols, 2 complementary systematic reviews were recently conducted, providing a comprehensive evaluation of the impact of hard palate closure timing.^3,4^

These reviews examined velopharyngeal insufficiency at 5 years. Overall, early closure was favored over late closure, as it was associated with lower incidences of fistula, improved speech outcomes and fewer additional surgeries. However, these studies highlighted challenges in comparing speech outcomes due to variations in assessment methods.^5^ Furthermore, there was a substantial indication of nonreporting bias, with smaller studies rarely reporting poor speech results.^4^ Consequently, there remains limited evidence about the comparative effectiveness of different surgical protocols in terms of speech. Furthermore, long-term speech outcomes are scarce in the existing literature, contributing to the evidence gap.^5–11^

To address these limitations, this study focuses on speech outcomes in UCLP patients treated with 4 distinct surgical protocols over the past decades at Erasmus Medical Center Rotterdam (EMC) and University Medical Center Utrecht (UMCU), the Netherlands. These protocols share a common timeline for lip closure (∼3–6 mo), soft palate closure (∼9–12 mo), and alveolar bone grafting (∼8–12 y) but differ in the timing of hard palate closure (Fig. 1):

1. Late Delayed Hard Palate Closure Protocol (L-DHPCP): Hard palate closure is performed simultaneously with alveolar bone grafting at 8 to 12 years.
2. Early Delayed Hard Palate Closure Protocol (E-DHPCP): Hard palate closure is performed between soft palate closure and alveolar bone grafting (∼2–8 y).
3. One-Stage Palatoplasty Protocol (OSPP): Simultaneous closure of the hard and soft palate at 9 to 12 months.
4. Oslo Protocol (OP): Closure of the anterior part of the hard palate using a vomer flap, performed with lip closure at 3 to 6 months, with the remainder of the palate closed at 9 to 12 months.

**FIGURE 1:**
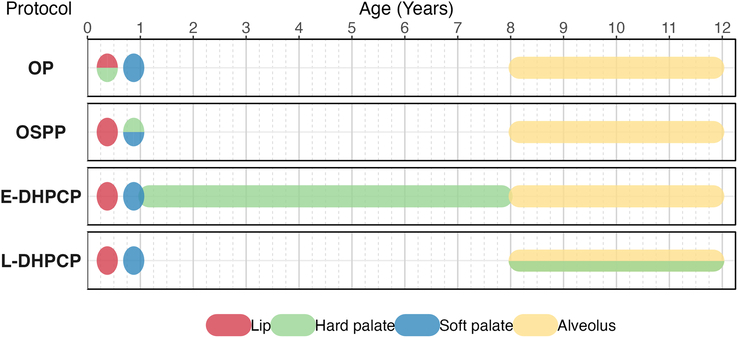
Protocol overview

This study aims to evaluate and compare speech outcomes, the frequency of speech-enhancing surgeries, and the presence of oronasal fistulas in separate cross-sectional cohorts of UCLP patients assessed at 5, 12, or 22 years of age. Specifically, it examines the impact of the timing of hard palate closure on both clinical and patient-reported speech outcomes. The findings will contribute to the body of evidence on the role of timing of hard palate closure in speech outcomes and support the pursuit of optimal surgical protocols to improve treatment for UCLP patients.

## METHODS

### Study Population

Dutch patients with UCLP who underwent speech assessment at EMC between February 2016 and November 2024, or at UMCU between February 2021 and November 2024, were eligible for inclusion. Within these periods, the International Consortium for Health Outcomes Measurement (ICHOM) Standard Set for Cleft Lip and Palate^12^ was used for the prospective follow-up during standard treatment (Ethical board approval no. METC-395478). Only UCLP patients with a complete cleft of the hard and soft palate were included. However, patients with incomplete cleft lips (ie, Simonart bands) were still eligible for inclusion, considering the minimal influence on speech outcomes. Patients with associated syndromes or genetic conditions, cognitive or developmental disabilities, or a late presentation were excluded. A late presentation was defined as patients who underwent soft palate closure beyond the age of 18 months, for instance, due to adoption. Furthermore, patients following different surgical protocols than those of interest, patients without complete information on their received surgical protocol, and patients without speech evaluation were also excluded.

Patients were included if they had undergone speech assessment at one of the predefined ages during the study period. As such, the study design was cross-sectional, and patients were not necessarily followed across multiple time points.

### Data Collection and Outcome Assessment

Speech was evaluated at 5, 12, and 22 years using the ICHOM Standard Set.^12^ The ICHOM is a set of outcome measures designed to evaluate health outcomes that matter most to cleft patients, including speech, appearance, and quality of life. Depending on the patient’s age, both clinical and patient-reported or parent-reported outcome measures (PROMs) were collected (Supplemental Table 1, Supplemental Digital Content 1, http://links.lww.com/SCS/I483). Clinical outcome measures related to speech were assessed by 1 of 6 specialized speech-language pathologists (SLP) in one-on-one sessions with the patient. Speech assessment included spontaneous speech and a restricted (Dutch) word list, conducted in a standardized setting and recorded on video for further analysis. The same Dutch word list was used in both centers and is valid for assessing all consonants. PROMs were completed by the patient and/or parents either at home or during their visit to the cleft team.

In addition to the outcome measures, timing and sequence of the primary surgeries for UCLP treatment (eg, lip, hard and soft palate, and alveolar closure) were retrospectively collected from medical records to determine the surgical protocol followed. It was not possible to reliably gather the specific surgical techniques applied to each patient retrospectively, and techniques were therefore not directly taken into account in this study. However, in both centers, historically, the Perko technique was used for soft palate closure, and the Sommerlad technique is now standard practice.^7,13^ For hard palate closure, the von Langenbeck technique (with or without relaxing incisions) or vomerplasty was used. In patients following E-DHPCP or L-DHPCP, no speech plates were used before hard palate closure.

### Clinical Outcome Measures

The Percentage of Consonants Correct (PCC) is a measure to assess articulatory proficiency based on the (in)correct production of target sounds from a restricted word list. The score, ranging from 0% to 100%, was calculated by dividing the number of correct consonants by the total number of elicited consonants. Active speech characteristics (eg, articulatory and phonological errors), distorted s-sounds and passive cleft distortions (nasal emission, nasal turbulence, and weak pressure consonants) where scored as incorrect, in accordance with the local scoring guidelines used at both centers. Scores ranging from 85% to 100% indicating mild to no speech problems; 65% to 85% mild to moderate problems; 50% to 65% moderate to severe problems; and <50% severe problems.^14^ It can be reliably used to evaluate the speech of cleft patients when performed by experienced clinicians.^15^

The Velopharyngeal Competence rate (VPC-R) is an overall assessment of velopharyngeal function (ie, hypernasality and nasal air leakage) and classifies patients into 3 categories: competent, marginally incompetent, and incompetent. Incompetent: when significant problems with VPC are observed, indicating a need for surgical intervention or further speech therapy. Marginally incompetent: when there is clinical evidence of only minor issues with competence. Patients are assigned to these categories based on the entire speech assessment (including single words, spontaneous speech). Previous research has demonstrated the reliability of VPC as a tool for clinical follow-up and research.^16^

The presence of oronasal fistulas at the time of speech assessment was recorded according to the Pittsburgh classification system.^17^ Fistulas were defined as an unintentional connection between the nasal and oral cavity, present at least 3 months after surgery. Only fistulas in parts of the cleft that had been previously operated on were considered (ie, residual open hard palates and alveolar clefts were not classified as fistulas).

The frequency and total number of speech-enhancing surgeries at the different time points were documented. The decision to perform additional surgery was made following a multidisciplinary evaluation of speech by the SLP and plastic surgeon.

### Patient-Reported Outcome Measures

The Intelligibility in Context Scale (ICS) is a 7-item parent-reported outcome measure primarily designed for children aged 4 to 6 years.^18^ It is completed by the parent and evaluates how well a child’s speech is understood by various communication partners, with mean scores ranging from 1.00 (low intelligibility) to 5.00 (high intelligibility).

The CLEFT-Q is a patient-reported outcome measure assessing intelligibility and quality of life in cleft patients from the age of 8.^19–21^ It comprises 13 scales covering several domains, including speech, which is evaluated through 2 distinct scales: speech function and speech distress. For both scales, lower scores indicate worse outcomes (range: 0–100). The speech function scale focuses on the functional aspects of speech difficulties, such as the ability to articulate certain letters or words.^20^ The speech distress scale addresses the psychosocial aspects of speech difficulties, such as feelings of nervousness or frustration.^20^

### Statistical Analysis

Statistical analyses were performed to compare outcomes across surgical protocols. Unadjusted comparisons for each outcome measure and patient characteristic were conducted using the Kruskal-Wallis *H* test for continuous or ordinal variables and the Fisher exact test for nominal variables. These analyses were carried out separately for each timepoint.

For speech-related outcomes, including VPC, PCC, ICS, and the CLEFT-Q speech distress and speech function scales, ordinal logistic regression models were fitted to evaluate differences between the surgical protocols. Ordinal logistic regression was chosen because the outcomes are ordinal and are either limited to a few categories (ie, VPC) or truncated within a specific range (ie, PCC, ICS, and CLEFT-Q scales). The models were adjusted for sex and age at the time of assessment.

When statistically significant differences were identified in the composite scores of PROMs, additional regression models were fitted at the level of individual questions to explore these differences in more detail. To account for multiple comparisons in these question-level analyses, the Bonferroni correction was applied, adjusting for the total number of comparisons made.

The results of the ordinal logistic regression models were presented as odds ratios (ORs) with 95% CIs, calculated using the profile likelihood method. For PCC, the regression model was fitted using the original scores; however, in the effect plots, these scores were categorized as described before to improve visualization and interpretability. Since no standardized categorizations are available for PROM scores, these outcomes were visualized in effect plots using their original scores.

All statistical analyses and visualizations were conducted using R statistical software (version 4.3.2).

## RESULTS

**FIGURE 2:**
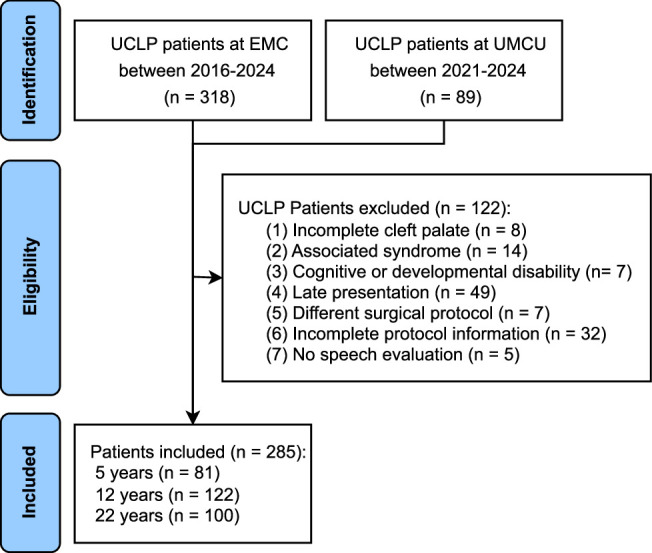
Flowchart of the patient selection process

A total of 407 UCLP patients treated at the EMC and UMCU were screened for inclusion, of whom 122 were excluded (Fig. 2). Ultimately, 285 patients were included, the majority of whom were male (N=207, 72.6%) (Supplemental Table 2, Supplemental Digital Content 1, http://links.lww.com/SCS/I483). Speech evaluations were available for 81 patients at 5 years, 122 at 12 years, and 100 at 22 years of age. Most patients were assessed at a single time point, with a few measured at 2 time points.

### 5-Year-Olds

**FIGURE 3:**
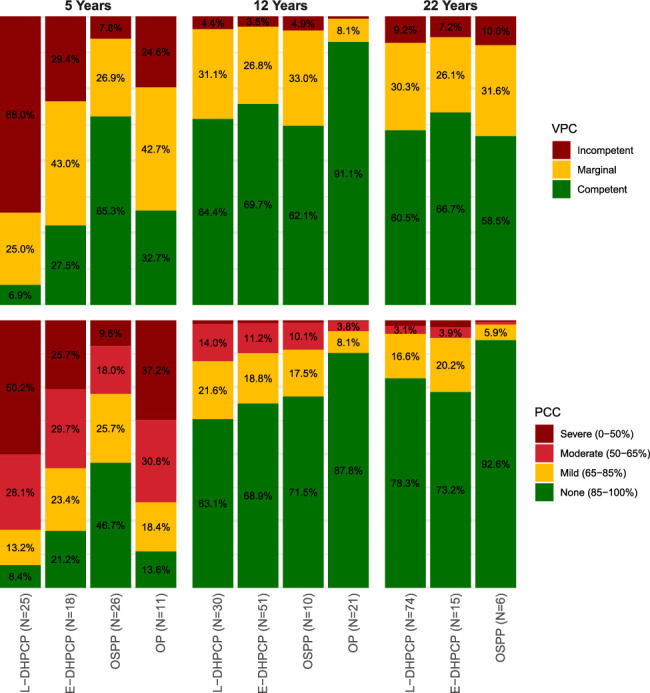
Effect plots of ordinal regression model results for velopharyngeal competence (VPC) and percentage of consonants correct (PCC) outcomes in males at exactly 5, 12, and 22 years.

At age 5, significant differences in speech outcomes were observed between the surgical protocols (Supplemental Table 3, Supplemental Digital Content 1, http://links.lww.com/SCS/I483). The odds of achieving competent VPC and higher PCC scores progressively increased with earlier hard palate closure (Fig. 3). Patients treated according to the E-DHPCP with a closed hard palate demonstrated significantly better results than those treated according to the L-DHPCP with an open hard palate (VPC: *P*=0.009; PCC: *P*=0.050). Furthermore, patients treated with the OSPP showed significantly better outcomes compared with both the L-DHPCP (VPC: *P*<0.001; PCC: *P*<0.001) and the E-DHPCP (VPC: *P*=0.011; PCC: *P*=0.046). While the sample size for OP was limited (N=11), results were generally comparable to the E-DHPCP and better than the L-DHPCP, particularly for VPC (*P*=0.016). However, OP scored significantly worse than OSPP in terms of PCC (*P*=0.016).

**FIGURE 4:**
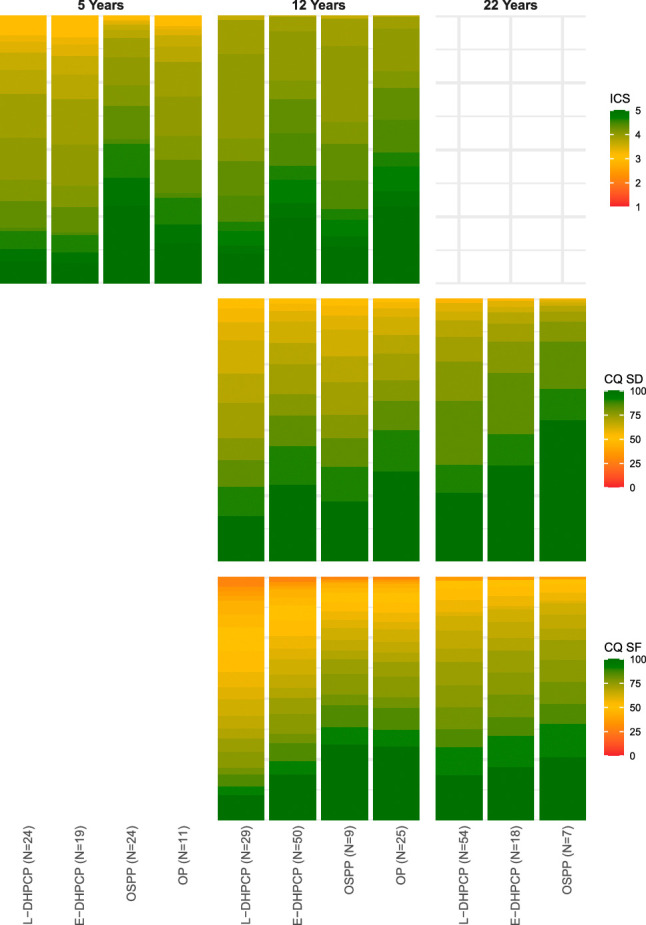
Effect plots of ordinal regression model results for Intelligibility in Context Scale (ICS), the CLEFT-Q speech distress scale (CQ-SD) and CLEFT-Q speech function scale (CQ-SF) outcomes in males at exactly 5, 12, and 22 years.

The ICS scores were highest with the OSPP and were significantly better than with the L-DHPCP (*P*=0.005) and the E-DHPCP (*P*=0.008). Question-level analysis revealed significant improvements in speech intelligibility for communication with friends (Q4: *P*=0.002) and other acquaintances (Q5: *P*=0.004) when comparing OSPP to L-DHPCP (Fig. 4, Supplemental Digital Content 2).

The frequency of speech-enhancing surgeries was highest with the E-DHPCP (52.6%) compared with the other protocols (*P*<0.001). In addition, the prevalence of oronasal fistulas was highest with the E-DHPCP (26.3%) and OSPP (26.9%), with no fistulas reported with the L-DHPCP (*P*=0.007). However, fistulas in the hard palate were not considered in the L-DHPCP, as hard palate closure had not yet been performed. Most fistulas were located in the hard palate (N=9, 69.2%).

### 12-Year-Olds

At 12 years of age, differences in speech outcomes between protocols were less pronounced (Supplemental Table 4, Supplemental Digital Content 1, http://links.lww.com/SCS/I483). The majority of patients across the protocols demonstrated competent VPC and PCC scores above 85%, indicating mild to no speech problems. Nevertheless, patients treated with the OP showed significantly higher odds of achieving competent VPC compared with the L-DHPCP (*P*=0.038). Similarly, PCC scores were significantly higher with the OP compared with both the L-DHPCP (*P*=0.009) and E-DHPCP (*P*=0.020). While the sample size for OSPP was limited (N=11), its results were generally consistent with the trends observed among the other protocols.

The ICS scores were significantly higher with the E-DHPCP (*P*=0.019) and OP (*P*=0.018), compared with the L-DHPCP. Question-level analysis revealed better speech intelligibility for communication with acquaintances (Q5: *P*=0.002) for OP compared with L-DHPCP. In addition, both E-DHPCP (*P*=0.005) and OP (*P*=0.003) demonstrated significantly better speech intelligibility for communication with strangers than L-DHPCP (Q7). The OP was also associated with significantly higher scores on the CLEFT-Q speech function scale compared with L-DHPCP (*P*=0.009). The question-level analysis highlighted fewer problems with speech intelligibility on the phone (Q6: *P*=0.002) and in person (Q7: *P*<0.001) for OP. Furthermore, patients treated with L-DHPCP have to repeat themselves significantly more often to be understood compared with those treated with OP (Q9: *P*=0.002).

The frequency of speech-enhancing surgeries was highest with the OP (60.7%) and E-DHPCP (50.9%) compared with the other protocols (*P*<0.001). Similarly, the prevalence of oronasal fistulas during speech assessment was highest with the E-DHPCP (7.5%) and OP (7.1%), with most fistulas (N=5, 71.4%) located in the hard palate.

### 22-Year-Olds

At 22 years of age, no significant differences in outcomes were observed between the protocols (Supplemental Table 5, Supplemental Digital Content 1, http://links.lww.com/SCS/I483). The majority of patients exhibited competent VPC and PCC scores above 85%, and speech outcomes were largely comparable across the protocols. However, the sample size was small, particularly for OSPP (N=7), and no patients treated with OP were available at this time point. The frequencies of speech-enhancing surgeries and the prevalence of oronasal fistulas were comparable between the L-DHPCP and E-DHPCP. Most fistulas (N=3, 60.0%) were located in the hard palate.

## DISCUSSION

This study compared speech outcomes in UCLP patients treated with protocols employing early (OSPP and OP) versus delayed (L-DHPCP and E-DHPCP) hard palate closure. The results demonstrated that protocols with early hard palate closure generally yielded more favorable speech results than those with delayed closure. However, some deviations were noted, likely attributable to limited sample sizes, particularly for OP at 5 and 22 years and OSPP at 12 years.

Overall, differences in speech outcome were most pronounced at 5 years, with OSPP and OP showing superior results across all primary outcome measures, including VPC, PCC, and ICS. At 12 years, OP and OSPP emerged as the protocols associated with the most favorable outcomes, while the differences were less pronounced. And even though OP and E-DHPCP had similarly high rates of speech-enhancing surgeries at this time point, minimizing potential confounding, speech outcomes for OP were still better. By 22 years, no significant differences in speech outcomes were observed between the available protocols (L-DHPCP, E-DHPCP, and OSPP), although persistent challenges with VPC and PCC remained for a considerable number of patients treated with L-DHPCP and E-DHPCP. However, due to the limited number of patients in the early repair group, these findings should be interpreted with caution.

These findings align with several previous studies indicating that early hard palate closure leads to better speech outcomes in the short term.^2,11,22,23^ Moreover, the Scandcleft trial found that delayed hard palate closure was associated with articulation errors that could impose a long-term psychosocial handicap. The trial concluded that extensive speech therapy did not effectively resolve these articulation errors, regardless of the frequency of sessions.^24^ In line with these findings, our results at 22 years suggest a persistence of speech problems, highlighting the importance of identifying optimal surgical protocols to minimize the need for prolonged and potentially ineffective speech therapy. It is particularly important given the global shortage of SLPs and the burden that frequent therapy sessions impose on patients and their families.

Given the findings of this study and the results of recent literature reviews, there is no compelling argument for delayed hard palate closure in nonsyndromic UCLP patients.^3,4^ Not one study has demonstrated improved maxillofacial growth in the long term by delaying hard palate closure,^3,4^ and worse speech outcomes have been repeatedly reported in association with delayed closure.^2,11,22–24^ As such, protocols employing early hard palate closure, such as OSPP and OP, should be prioritized in clinical practice for nonsyndromic UCLP patients.

Despite the advantages of early hard palate closure, it is notable that acceptable long-term outcomes were achieved for many patients with DHPCPs, albeit at the cost of additional speech-enhancing surgeries. These findings suggest that while early closure protocols should be the standard of care to optimize speech development and reduce the burden of care, delayed protocols may still be viable in specific clinical scenarios, such as for patients with associated syndromes and respiratory challenges (eg, Treacher Collins syndrome). Future studies should explore how patient-specific factors, such as cleft width and syndromic diagnoses, should influence the choice of protocol.

Several limitations of this study should be acknowledged. First, speech assessment was conducted by only 1 of 6 specialized SLPs, limiting assessment of inter-rater and intrarater reliability and introducing potential subjectivity. This was partially mitigated through ICHOM-based standardization of speech assessment and biannual calibration sessions, which significantly improve reliability.^25^ Second, interpretation of PCC scores requires some caution, particularly for younger children. In the PCC-R, as per the ICHOM methodology,^12^ distortions are scored as correct, which may lead to underestimation of cleft-specific speech characteristics. Moreover, PCC-R assigns equal weight to all types of errors (eg, emissions, substitutions, and distortions), although these may differ in clinical relevance. For instance, certain distortions may be developmentally appropriate during the early stages of speech development, whereas omissions and substitutions are not. In our centers, distortions are scored as incorrect, in line with our local guidelines. However, such differences in PCC scoring across centers further complicate outcome comparisons and highlight the need for a consistent, cleft-patient-specific approach.^14^ However, as this limitation applies to all groups in this study, systematic bias in comparisons is unlikely. Third, this study was unable to account for initial cleft width and surgical technique for soft palate closure, as treatment information was collected retrospectively. These factors are critical for understanding speech outcomes and should ideally be included in future studies. Fourth, the cross-sectional design of this study limits conclusions about the speech development of individual patients over time, as observed differences may reflect cohort variation rather than true progression. Lastly, the relatively small sample sizes for certain groups, particularly OP at 5 years and OSPP at 12 years may have limited the statistical power of our analyses.

Fifth, data on the timing, content, and duration of speech therapy were not available, as therapy is provided by community-based speech-language pathologists outside of the cleft teams. This decentralized model of care limits the ability to analyse the relationship between specific therapy regimens and speech outcomes. Future studies could consider incorporating standardized reporting tools to capture therapy-related data across centers.

To further advance research on cleft speech, future studies should include large, multicentre cohorts with prospective data collection. Since the effect of hard palate closure timing has been largely clarified, these studies should rather focus on identifying the most effective soft palate closure techniques and tailoring them to individual patients based on characteristics such as cleft width. Addressing the apparent nonreporting bias in existing literature is also critical, as it undermines the reliability of current evidence.^4^ Multicentre studies can reduce this bias by anonymising data across institutions, but centers must commit to transparent reporting of all outcomes, both positive and negative. Without this, progress toward optimal treatment protocols will remain hindered.

## CONCLUSION

In conclusion, this study highlights the benefits of early hard palate closure protocols, such as OSPP and OP, in achieving more favorable short-term and intermediate speech outcomes compared with DHPCPs. Furthermore, the persistent speech difficulties observed at 22 years underscore the importance of identifying optimal surgical protocols and tailoring them to the patient’s needs. On the basis of these findings and the existing body of evidence, protocols with early hard palate closure should be prioritized in nonsyndromic UCLP patients to prevent persistent speech errors and minimize the burden of (speech-enhancing) surgeries and speech therapy.

## Supplementary Material

SUPPLEMENTARY MATERIAL
