# Supplementary material for: Long-Term Speech Outcomes in Unilateral Cleft Lip and Palate: A Comparative Study of Early and Delayed Hard Palate Closure
Source: J Craniofac Surg. 2025 Sep 23;36(8):2823–8. doi: 10.1097/SCS.0000000000011975 (PMC12537028; doi:10.1097/SCS.0000000000011975)
Supplement: SUPPLEMENTARY MATERIAL [file scs-36-02823-s001.docx]

Supplemental Table 1 – Speech-related outcome measures from the ICHOM Standard Set for Cleft Lip and Palate per time point.

|  | **Time point (Years)** | | |
| --- | --- | --- | --- |
|  | **5** | **12** | **22** |
| **Clinical outcome measures** | | | |
| Velopharyngeal Competence (VPC) | X | X | X |
| Percentage of Consonants Correct (PCC) | X | X | X |
| Pure Tone Average (PTA) | X | X |  |
| Presence of oronasal fistulas | X | X | X |
| Number of previous speech-enhancing surgeries | X | X | X |
| **PROMs** | | | |
| Intelligibility in Context Scale (ICS) | X | X |  |
| CLEFT-Q – Speech Distress scale |  | X | X |
| CLEFT-Q – Speech Function scale |  | X | X |

Supplemental Table 2 – Patient characteristics stratified by surgical protocol

|  | **L-DHPCP (N=106, 37.2%)** | **E-DHPCP (N=97, 34.0%)** | **OSPP (N=43, 15.1%)** | **OP (N=39, 13.7%)** | **Total  (N=285)** |
| --- | --- | --- | --- | --- | --- |
| **Sex** |  |  |  |  |  |
| Female | 36 (34.0%) | 20 (20.6%) | 10 (23.3%) | 12 (30.8%) | 78 (27.4%) |
| Male | 70 (66.0%) | 77 (79.4%) | 33 (76.7%) | 27 (69.2%) | 207 (72.6%) |
|  |  |  |  |  |  |
| **Mean age at surgery in years (SD)** | | | | | |
| Lip closure | 0.36 (0.131) | 0.39 (0.217) | 0.37 (0.158) | 0.40 (0.227) | 0.37 (0.181) |
| Soft palate closure | 0.93 (0.159) | 0.89 (0.186) | 0.97 (0.246) | 0.85 (0.257) | 0.91 (0.200) |
| Hard palate closure | 10.70 (1.080) | 4.93 (1.610) | 0.97 (0.246) | 0.40 (0.227) | 5.92 (4.240) |
| ABG | 10.70 (1.080) | 10.20 (0.990) | 10.50 (1.130) | 10.00 (1.200) | 10.40 (1.090) |
|  |  |  |  |  |  |
| **Centre** |  |  |  |  |  |
| EMC | 106 (100%) | 88 (90.7%) | 37 (86.0%) | 4 (10.3%) | 235 (82.5%) |
| UMCU | 0 (0%) | 9 (9.3%) | 6 (14.0%) | 35 (89.7%) | 50 (17.5%) |
|  |  |  |  |  |  |

L-DHPCP = Late Delayed Hard Palate Closure Protocol; E-DHPCP = Early Delayed Hard Palate Closure Protocol; OSPP = One-Stage Palatoplasty Protocol; OP = Oslo Protocol; ABG = Alveolar Bone Grafting; EMC = Erasmus Medical Centre; UMCU = University Medical Centre Utrecht

Supplemental Table 3 – Results at 5 years of age (N = 81)

|  | **L-DHPCP***  **(N=25)** | **E-DHPCP***  **(N=19)** | **OSPP**  **(N=26)** | **OP (N=11)** | **ρ** |
| --- | --- | --- | --- | --- | --- |
| ***Age at measurement (in years)*** | | |  |  | |
| Mean (SD) | 4.4 (0.758) | 4.7 (0.881) | 4.2 (0.401) | 5.1 (1.280) | 0.051^1^ |
| Median [Min, Max] | 4.1 [3.7, 6.1] | 4.2 [4.0, 6.2] | 4.1 [4.0, 6.1] | 5.0 [3.6, 7.3] |  |
| ***Sex*** |  |  |  |  |  |
| Female (%) | 4 (16.0%) | 4 (21.1%) | 6 (23.1%) | 2 (18.2%) | 0.958^2^ |
| Male (%) | 21 (84.0%) | 15 (78.9%) | 20 (76.9%) | 9 (81.8%) |  |
| ***Velopharyngeal Competence (VPC)*** | | |  |  | |
| Competent | 2 (8.0%) | 3 (15.8%) | 16 (61.5%) | 3 (27.3%) | OLR |
| Marginally incompetent | 5 (20.0%) | 10 (52.6%) | 6 (23.1%) | 5 (45.5%) |  |
| Incompetent | 18 (72.0%) | 5 (26.3%) | 4 (15.4%) | 3 (27.3%) |  |
| *Missing* | 0 (0%) | 1 (5.3%) | 0 (0%) | 0 (0%) |  |
| ***Percentage of Consonants Correct (PCC)*** | | |  |  | |
| Mean (SD) | 41.7 (22.0) | 60.9 (25.6) | 72.5 (27.5) | 51.3 (27.7) | OLR |
| Median [Min, Max] | 52.0 [5.0, 70.0] | 61.0 [9.0, 97.0] | 80.5 [12.0, 100] | 57.0 [14.0, 88.0] |  |
| Missing | 0 (0%) | 1 (5.3%) | 0 (0%) | 0 (0%) |  |
| ***Intelligibility in Context Scale (ICS)*** | | |  |  | |
| Mean (SD) | 3.9 (0.509) | 3.9 (0.481) | 4.3 (0.595) | 4.2 (0.550) | OLR |
| Median [Min, Max] | 3.9 [3.1, 5.0] | 3.9 [3.1, 4.9] | 4.3 [3.1, 5.0] | 4.1 [3.1, 5.0] |  |
| Missing | 1 (4.0%) | 0 (0%) | 2 (7.7%) | 0 (0%) |  |
| ***Pure Tone Average*** | | |  |  | |
| Mean (SD) | 20.5 (14.6) | 15.6 (11.2) | 19.1 (9.51) | 29.2 (12.2) | 0.168^1^ |
| Median [Min, Max] | 16.5 [3.50, 51.5] | 11.5 [5.0, 48.0] | 15.0 [8.0, 37.5] | 29.0 [13.5, 45.5] |  |
| Missing | 5 (20.0%) | 5 (26.3%) | 11 (42.3%) | 6 (54.5%) |  |
| ***Speech-enhancing surgery*** | | |  |  | |
| Total | 2 (8.0%) | 10 (52.6%) | 1 (3.8%) | 1 (9.1%) | **<0.001**^2^ |
| One surgery | 2 (8.0%) | 9 (47.4%) | 1 (3.8%) | 1 (9.1%) |  |
| Two surgeries | 0 (0%) | 1 (5.3%) | 0 (0%) | 0 (0%) |  |
| ***Oronasal fistula*** | | |  |  | |
| *Total* | 0 (0%) | 5 (26.3%) | 7 (26.9%) | 1 (9.1%) | **0.001^2^** |
| Type II: Soft palate | 0 (0%) | 0 (0%) | 1 (3.8%) | 0 (0%) |  |
| Type III: Junction SP/HP | - | 1 (5.3%) | 2 (7.7%) | 0 (0%) |  |
| Type IV: Hard palate | - | 4 (21.1%) | 4 (15.4%) | 1 (9.1%) |  |

L-DHPCP = Late Delayed Hard Palate Closure Protocol; E-DHPCP = Early Delayed Hard Palate Closure Protocol; OSPP = One-Stage Palatoplasty Protocol; OP = Oslo Protocol; SP = soft palate; HP = hard palate;

*Patients in L-DHPCP had an open hard palate, while in E-DHPCP the hard palate was closed at this time point;

^1^Kruskal Wallis H test or ^2^Fisher Exact test;

OLR = Ordinal logistic regression (Supplementary Digital Content 1)

Supplemental Table 4 – Results at 12 years of age (N = 122)

|  | **L-DHPCP (N=30)** | **E-DHPCP (N=53)** | **OSPP (N=11)** | **OP**  **(N=28)** | **ρ** |
| --- | --- | --- | --- | --- | --- |
| ***Age at measurement (in years)*** | | |  |  |  |
| Mean (SD) | 12.2 (0.568) | 12.2 (0.157) | 12.0 (0.466) | 12.2 (0.701) | 0.504^1^ |
| Median [Min, Max] | 12.1 [11.7, 15.1] | 12.1 [12.0, 12.7] | 12.1 [10.9, 12.7] | 12.3 [10.7, 13.7] |  |
| ***Sex*** | | |  |  |  |
| Female (%) | 10 (33.3%) | 12 (22.6%) | 2 (18.2%) | 10 (35.7%) | 0.490^2^ |
| Male (%) | 20 (66.7%) | 41 (77.4%) | 9 (81.8%) | 18 (64.3%) |  |
| ***Velopharyngeal Competence (VPC)*** | | |  |  |  |
| Competent | 19 (63.3%) | 35 (66.0%) | 6 (54.5%) | 19 (67.9%) | OLR |
| Marginally incompetent | 9 (30.0%) | 14 (26.4%) | 4 (36.4%) | 2 (7.1%) |  |
| Incompetent | 2 (6.7%) | 2 (3.8%) | 0 (0%) | 0 (0%) |  |
| *Missing* | 0 (0%) | 2 (3.8%) | 1 (9.1%) | 7 (25.0%) |  |
| ***Percentage of Consonants Correct (PCC)*** | | |  |  |  |
| Mean (SD) | 85.3 (14.7) | 87.3 (15.2) | 87.1 (16.3) | 97.1 (4.31) | OLR |
| Median [Min, Max] | 90.0 [52.0, 100] | 92.5 [48.0, 100] | 95.5 [58.0, 100] | 100 [87.0, 100] |  |
| Missing | 1 (3.3%) | 3 (5.7%) | 1 (9.1%) | 9 (32.1%) |  |
| ***Intelligibility in Context Scale (ICS)*** | | |  |  |  |
| Mean (SD) | 4.3 (0.385) | 4.6 (0.400) | 4.3 (0.490) | 4.6 (0.427) | OLR |
| Median [Min, Max] | 4.3 [3.9, 5.0] | 4.6 [3.9, 5.0] | 4.3 [3.6, 5.0] | 4.7 [4.0, 5.0] |  |
| Missing | 1 (3.3%) | 3 (5.7%) | 2 (18.2%) | 3 (10.7%) |  |
| ***CLEFT-Q Speech Distress*** | | |  |  |  |
| Mean (SD) | 78.4 (16.2) | 83.0 (15.6) | 80.0 (16.0) | 86.2 (12.8) | OLR |
| Median [Min, Max] | 77.0 [56.0, 100] | 83.0 [49.0, 100] | 74.5 [63.0, 100] | 90.0 [60.0, 100] |  |
| Missing | 9 (30.0%) | 10 (18.9%) | 1 (9.1%) | 0 (0%) |  |
| ***CLEFT-Q Speech Function*** | | |  |  |  |
| Mean (SD) | 65.4 (17.0) | 72.1 (20.7) | 77.0 (26.8) | 80.1 (17.5) | OLR |
| Median [Min, Max] | 63.0 [44.0, 100] | 73.0 [28.0, 100] | 86.5 [28.0, 100] | 82.5 [47.0, 100] |  |
| Missing | 9 (30.0%) | 9 (17.0%) | 1 (9.1%) | 0 (0%) |  |
| ***Pure Tone Average*** | | |  |  |  |
| Mean (SD) | 10.7 (8.90) | 9.9 (7.35) | 13.0 (9.20) | 13.5 (8.62) | 0.468^1^ |
| Median [Min, Max] | 9.50 [1.0, 34.0] | 9.00 [0, 35.5] | 8.00 [6.0, 25.5] | 9.75 [4.5, 34.0] |  |
| Missing | 7 (23.3%) | 10 (18.9%) | 5 (45.5%) | 14 (50.0%) |  |
| ***Speech-enhancing surgery*** | | |  |  |  |
| Total | 5 (16.7%) | 27 (50.9%) | 2 (18.2%) | 17 (60.7%) | **<0.001^2^** |
| One surgery | 4 (13.3%) | 21 (39.6%) | 1 (9.1%) | 15 (53.6%) |  |
| Two surgeries | 1 (3.3%) | 5 (9.4%) | 0 (0%) | 1 (3.6%) |  |
| Three surgeries | 0 (0%) | 1 (1.9%) | 1 (9.1%) | 1 (3.6%) |  |
| ***Oronasal fistula*** | | |  |  |  |
| *Total* | 1 (3.3%) | 4 (7.5%) | 0 (0%) | 2 (7.1%) | 0.889^2^ |
| Type III: Junction SP/HP | 0 (0%) | 2 (3.8%) | 0 (0%) | 0 (0%) |  |
| Type IV: Hard palate | 1 (3.3%) | 2 (3.8%) | 0 (0%) | 2 (7.1%) |  |

L-DHPCP = Late Delayed Hard Palate Closure Protocol; E-DHPCP = Early Delayed Hard Palate Closure Protocol; OSPP = One-Stage Palatoplasty Protocol; OP = Oslo Protocol; SP = soft palate; HP = hard palate;

^1^Kruskal Wallis H test or ^2^Fisher Exact test;

OLR = Ordinal logistic regression (Supplementary Digital Content 1)

Supplemental Table 5 – Outcomes at 22 years of age (N = 100)

|  | **L-DHPCP (N=74)** | **E-DHPCP (N=19)** | **OSPP (N=7)** | **ρ** |
| --- | --- | --- | --- | --- |
| ***Age at measurement (in years)*** | | |  |  |
| Mean (SD) | 22.2 (0.231) | 22.2 (0.480) | 22.0 (0.335) | 0.275^1^ |
| Median [Min, Max] | 22.1 [21.5, 23.1] | 22.2 [21.0, 23.1] | 22.0 [21.3, 22.4] |  |
| ***Sex*** | | |  |  |
| Male (%) | 26 (35.1%) | 6 (31.6%) | 2 (28.6%) | 1.000^2^ |
| Female (%) | 48 (64.9%) | 13 (68.4%) | 5 (71.4%) |  |
| ***Velopharyngeal Competence (VPC)*** | | |  |  |
| Competent | 43 (58.1%) | 10 (52.6%) | 3 (42.9%) | OLR |
| Marginally incompetent | 21 (28.4%) | 4 (21.1%) | 3 (42.9%) |  |
| Incompetent | 8 (10.8%) | 1 (5.3%) | 0 (0%) |  |
| *Missing* | 2 (2.7%) | 4 (21.1%) | 1 (14.3%) |  |
| ***Percentage of Consonants Correct (PCC)*** | | |  |  |
| Mean (SD) | 91.6 (14.9) | 89.4 (14.0) | 95.0 (12.2) | OLR |
| Median [Min, Max] | 100 [15.0, 100] | 100 [61.0, 100] | 100 [70.0, 100] |  |
| Missing | 3 (4.1%) | 4 (21.1%) | 1 (14.3%) |  |
| ***CLEFT-Q Speech Distress*** | | |  |  |
| Mean (SD) | 84.2 (13.5) | 87.7 (11.8) | 92.3 (7.76) | OLR |
| Median [Min, Max] | 83.0 [46.0, 100] | 86.5 [63.0, 100] | 90.0 [83.0, 100] |  |
| Missing | 20 (27.0%) | 1 (5.3%) | 0 (0%) |  |
| ***CLEFT-Q Speech Function*** | | |  |  |
| Mean (SD) | 79.1 (16.3) | 80.9 (15.0) | 82.4 (18.0) | OLR |
| Median [Min, Max] | 76.0 [37.0, 100] | 80.0 [50.0, 100] | 85.0 [53.0, 100] |  |
| Missing | 20 (27.0%) | 1 (5.3%) | 0 (0%) |  |
| ***Speech-enhancing surgery*** | | |  |  |
| Total | 24 (32.4%) | 7 (36.8%) | 0 (0%) | 0.170^2^ |
| One surgery | 21 (28.4%) | 5 (26.3%) | 0 (0%) |  |
| Two surgeries | 3 (4.1%) | 2 (10.5%) | 0 (0%) |  |
| ***Oronasal fistula*** | | |  |  |
| *Total* | 4 (5.4%) | 0 (0%) | 1 (14.3%) | 0.234^2^ |
| Type II: Soft palate | 1 (1.4%) | 0 (0%) | 0 (0%) |  |
| Type IV: Hard palate | 2 (2.7%) | 0 (0%) | 1 (14.3%) |  |
| Type VII: Labial-alveolar | 1 (1.4%) | 0 (0%) | 0 (0%) |  |

L-DHPCP = Late Delayed Hard Palate Closure Protocol; E-DHPCP = Early Delayed Hard Palate Closure Protocol; OSPP = One-Stage Palatoplasty Protocol; OP = Oslo Protocol;

^1^Kruskal Wallis H test or ^2^Fisher Exact test;

OLR = Ordinal logistic regression (Supplementary Digital Content 1)
